# Left ventricular unloading during veno-arterial ECMO: a review of percutaneous and surgical unloading interventions

**DOI:** 10.1177/0267659118794112

**Published:** 2018-08-16

**Authors:** Dirk W. Donker, Daniel Brodie, José P.S. Henriques, Michael Broomé

**Affiliations:** 1 Department of Intensive Care Medicine, University Medical Center Utrecht, Utrecht University, Utrecht, The Netherlands; 2 Division of Pulmonary, Allergy and Critical Care Medicine, Columbia University College of Physicians and Surgeons/New York-Presbyterian Hospital, New York, NY, USA; 3 Department of Cardiology, Academic Medical Center, Amsterdam UMC, University of Amsterdam, Amsterdam, The Netherlands; 4 ECMO Department, Karolinska University Hospital, Stockholm, Sweden; 5 Anaesthesiology and Intensive Care, Department of Physiology and Pharmacology, Karolinska Institute, Stockholm, Sweden; 6 School of Technology and Health, Royal Institute of Technology, Stockholm, Sweden

**Keywords:** veno-arterial extracorporeal membrane oxygenation (VA ECMO), left ventricular unloading, cardiogenic shock, cardiovascular modeling, computer simulation

## Abstract

Short-term mechanical support by veno-arterial extracorporeal membrane oxygenation (VA ECMO) is more and more applied in patients with severe cardiogenic shock. A major shortcoming of VA ECMO is its variable, but inherent increase of left ventricular (LV) mechanical load, which may aggravate pulmonary edema and hamper cardiac recovery. In order to mitigate these negative sequelae of VA ECMO, different adjunct LV unloading interventions have gained a broad interest in recent years. Here, we review the whole spectrum of percutaneous and surgical techniques combined with VA ECMO reported to date.

## Introduction

The widespread use of veno-arterial extracorporeal membrane oxygenation (VA ECMO) in severe cardiogenic shock is importantly driven by its relative ease of implantation and immediate reversal of inadequate systemic perfusion.^1^ Yet, clinical management of VA ECMO remains challenging and requires mechanistic insights and careful monitoring of a proper balance between the circulatory needs and the cardiac condition of an individual patient.^2^ Although, VA ECMO allows full circulatory support to counteract a severe shock state, the continuous, extracorporeal blood flow and increased aortic pressure are opposed to the ejection of the native, failing heart.

**Figure 1.**
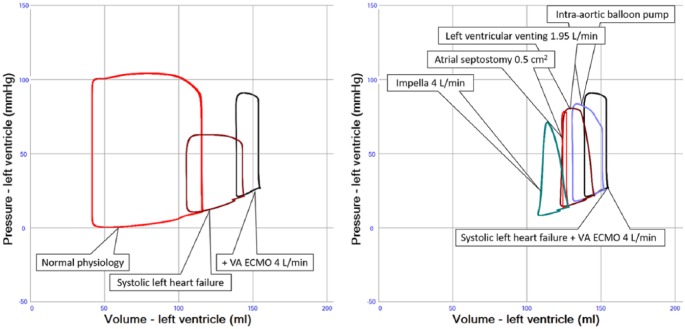
**a**. LV pressure-volume loops in normal physiology (red), severe systolic left heart failure (dark red) and when systemic circulation is supported by VA ECMO 4 L/min (black). Right shift of the loop indicates dilatation, which is worsened with VA ECMO, mainly due to an increase in afterload. **b**. LV pressure-volume loops in severe left heart failure supported by VA ECMO 4 L/min (black) in conjunction with different support modalities aimed at LV unloading. Intra-aortic balloon pumping (blue) results in increasing stroke volumes, with a modest unloading effect, while LV venting into the ECMO system (dark red) achieves better unloading. Atrial septostomy (red) achieves efficient LV unloading, but results in smaller stroke volumes, while the most effective unloading is seen with the Impella^®^ (green). For further details, see main text.

It is increasingly recognized that VA ECMO, especially peripheral VA ECMO, may significantly increase left ventricular (LV) afterload through retrograde infusion of arterialized blood into the descending aorta.^1–4^ As a consequence of this inherent limitation of VA ECMO, the myocardium is overloaded (Figure 1a) and the impaired LV dilates, while increased filling pressures and pulmonary edema ensue. All these adverse sequelae of VA ECMO not only significantly limit cardiac recovery, but also negatively impact on long-term prognosis.^5,6^

In order to minimize LV overload and support recovery of the failing heart during VA ECMO, different percutaneous and surgical adjunct interventions to VA ECMO have been proven to be useful in clinical practice, as recently also supported by comprehensive cardiovascular modeling and computer simulation.^2,7^

### Intra-aortic balloon pump combined with VA ECMO

The intra-aortic balloon pump (IABP) has been used as an adjunct to VA ECMO for almost two decades and in more than 2,500 patients reported in the literature (supplemental Table 1 and supplemental Figure 1) in order to create pulsatile flow and afterload reduction. In post-cardiotomy, post heart transplantation and medical VA ECMO indications, including refractory cardiac arrest, concomitant IABP has been considered to be an independent predictor of improved survival and facilitates weaning from VA ECMO.^8–12^ Therefore, some authors have advocated the use of an IABP for all patients on VA ECMO, although this strategy has not universally been agreed upon.^8,10,11^ Recent clinical data are conflicting and partly contrast this notion for post-cardiotomy failure, infarct-related shock and other clinical settings based on extensive analyses that do not unequivocally support the routine combination of VA ECMO and IABP as a clinical standard,^13–15^ whereas another recent analysis favors its application and suggests a survival benefit.^16^

Mechanistically, it has been shown that the combined use can enhance patients’ blood flow in the native coronary arteries as well as in bypass grafts, although this has been questioned in an experimental setting and may importantly depend on the VA ECMO configuration, cannulation sites and cannula direction.^17–19^ The IABP may also negatively impact on the spinal cord and cerebral blood flow during VA ECMO, especially when the native cardiac function is severely impaired, while experimental studies challenge this notion.^19–21^ Moreover, experimental work on the combined use has demonstrated an improved myocardial oxygen-supply-demand balance in central and peripheral VA ECMO,^22^ whereas a beneficial impact on the microcirculation seems to be lacking.^23,24^

From a theoretical perspective, the introduction of an IABP during VA ECMO could be expected to reduce the mean systemic impedance, systolic pressure and peak LV wall stress by 10-15%^25^ (Figure 1b). Therefore, adjunct use of the IABP may be indicated in a persistently non-ejecting LV during peripheral or central VA ECMO support, where LV thrombosis is impending and systolic afterload reduction may allow aortic valve opening and switching to alternative cardiac mechanical support is not desired or possible.^21,26-28^ Moreover, clinical studies focusing on beneficial effects of the adjunct IABP on LV filling pressures have reported reduced central venous pressure, pulmonary capillary wedge pressure (PCWP), smaller LV dimensions and less pulmonary edema on chest x-ray.^24,29,30^ This may not only have a beneficial effect on pulmonary edema, which is thought to complicate at least 30% of VA ECMO treatments, but translate into improved prognosis, even after successful bridge-to-bridge long-term LVAD strategies.^5,6,29^ In summary, the LV unloading effect of an IABP during VA ECMO is rather limited and the PCWP decrease has been reported to reach maximally 5 mmHg, which is supported by clinical experience and computer simulations (Supplemental data/file 2).^7,17,24^

### Percutaneous trans-aortic LV assist device Impella^®^ combined with VA ECMO

The Impella^®^ (Abiomed, Danvers, MA) is a trans-aortic LV assist device designed as a catheter-based, micro-axial impeller pump that provides continuous blood flow from the LV into the ascending aorta. The 2.5 device provides up to 2.5 L/min blood flow and is intended for percutaneous short-term mechanical support in cardiogenic shock,^31^ but may lack sufficient blood flow in patients presenting with profound cardiogenic shock, when multi-organ failure is impending and mechanical ventilation is required.^32,33^

The Impella^®^ 2.5 and the larger CP device have been reported in a number of adult cases (n=151 cases, supplemental Table 1 and supplemental Figure 1) as adjunct to peripheral VA ECMO in order to unload the LV,^34,35^ representing the second largest clinical experience as LV unloading intervention during VA ECMO following the IABP. All available Impella^®^ devices for LV support, i.e., 2.5, CP and the surgical 5.0 device have been combined with VA ECMO and revealed a clear reduction of right atrial pressure, PCWP, LV volumes and pulmonary edema in adults (Figure 1b) and, also, increased pulmonary blood flow and right-ventricular performance^34–36^ in children.^37^ Moreover, a large retrospective analysis suggested a survival benefit and improved bridging to recovery or additional support therapy when combining the Impella^®^ with VA ECMO.^35^ The Impella^®^ device should, therefore, be considered as a powerful LV unloading device during VA ECMO, which is also supported by simulation experiments, indicating a maximum reduction of PCWP of 10 mmHg and LV volume of 20% as a function of Impella^®^ flow (Supplemental data/file 2).^7^

### Atrial septostomy during VA ECMO

With an atrial septum bulging from left to right, as visualized on the echocardiogram and indicating higher left-sided than right-sided filling pressures, it is tempting to unload the LV by creating an atrial septal defect.^38–40^ The favorable effect of such a left-to-right shunt on LV decompression and LV loading conditions has been reported to be significant in neonates,^41^ children^38–40,42^ and adults,^40,43^ as supported by experimental data^7^ (Figure 1b). Yet, it can be technically difficult to create an appropriately sized defect, which may critically impact on LV unloading and potentially even result in a non-ejecting LV, cavity and aortic root thrombosis (Supplemental data/file 2). Alternatively, a dedicated percutaneous device can be used to create well-defined sizes of the atrial septal defect,^44,45^ allowing trans-catheter exchange of the device as well as replacement with a definite closure device when not needed any longer.^46^ It should be realized that atrial septostomy has been reported as one of the first LV unloading techniques during VA ECMO, but remains technically demanding and experience is largely confined to specialized centers and pediatric patients^41^ (supplemental Table 1 and supplemental Figure 1).

### Percutaneous trans-septal left atrial pulmonary artery and trans-aortic LV venting during VA ECMO

A percutaneous approach to vent the LV by cannulation via the interatrial septum has successfully been applied in children^42,45,47–49^ and adults^48,50–52^ and has, as the atrial septostomy, already been reported in the 1990s^53^ (supplemental Table 1 and supplemental Figure 1). It is understandable that the LV venting effect of a cannula in the left atrium is very comparable to an atrial septostomy, as described above. Yet, the blood flow drained to the venous side of the ECMO circuit importantly depends on the sizing of the cannula and tubing^48,51^ and flow can potentially be controlled by a separate pump and, also, temporarily be clamped during weaning. In practice, it seems that using a 22F cannula can offer a potent, yet variable degree of LV unloading with a PCWP reduction ranging between 4 mmHg and 17 mmHg.^52^ If the draining cannula is advanced through the mitral valve into a non-ejecting LV, ventricular drainage will assure circulation of blood in the LV cavity and decrease the risk of flow stagnation and cavity thrombosis. Alternatively, the percutaneous route can also be used to access the LV via a trans-aortic catheter, as proposed in experimental and clinical studies in a very limited number of cases via an axillary or femoral artery approach^54–56^ (supplemental Table 1 and supplemental Figure 1). An indirect technique using a trans-pulmonary artery catheter can be adopted for LV venting, which is based on experimental findings^57,58^ and clinical studies in children and adults.^59–61^ It is important to realize that pulmonary artery venting may, theoretically, result in decreased flow in the pulmonary circulation, resulting in ischemia of the lung and, therefore, implicates close monitoring of pulmonary artery flow and/or end-tidal CO_2_.^62^

### Direct surgical LV, LA and pulmonary artery venting during VA ECMO

A direct surgical approach to unloading the LV requires an apical vent or a venting cannula introduced via the right superior pulmonary vein or, exceptionally, the pulmonary artery, which requires sternotomy or thoracotomy, although minimally invasive approaches have been suggested.^42,63–67^ Although, the experience reported in the literature is relatively limited (supplemental Table 1 and supplemental Figure 1), this might not hold for the regular practice of the technique in selected cardiothoracic surgical centers.

Surgical venting techniques use larger-sized cannulae compared to percutaneous approaches, which, in turn, allow improved venous drainage and substantial LV unloading, as supported by clinical experimental data (Figure 1b)(supplemental Table 1 and supplemental Figure 1),^7^ yet remain to carry substantial risks of bleeding.^68^ In addition, patient care and mobilization in the ICU is challenging.^68^ Short-term use of venting post-cardiotomy as an adjunct to central arterial cannulation is supported by some authors, despite considerable mortality and complication rates.^42,67^

Alternatively, it has been proposed to anticipate temporary LV assist-device implantation upon initiation of VA ECMO, including adequate LV venting.^66,69^ In this sense, it can be considered to use a large-sized LV apical vent with a minimum of a 32-F drainage cannula. Venous drainage of the VA ECMO circuit can be accomplished by percutaneous venous drainage, while aortic access is obtained with a 10-mm Dacron graft on the ascending aorta. This configuration allows tailoring of LV venting and RV drainage, using adjustable clamps and blood flow meters mounted on the circuit tubing. When right ventricular and pulmonary function have recovered sufficiently, this VA ECMO setup can easily be converted at the bedside to a more sustainable temporary LV assist device by simply removing the drainage cannula in the femoral vein.^66,70^

## Practical implications for clinical decision making

Peripheral VA ECMO remains the fastest and most reliable method to institute systemic rescue perfusion in life-threatening cardiac low-output states.^1^ This extracorporeal support strategy provides immediate restitution of organ perfusion and oxygenation and, therefore, enables clinicians to establish a bridge to decision, recovery or alternative therapies in a variety of settings. Yet, it should be realized that outcome is less favorable beyond a few weeks of support due to inherent limitations and the invasiveness of VA ECMO.

Bridging cardiac failure with VA ECMO is challenging and the potential for short-term myocardial recovery will largely depend on the underlying disease and its individual manifestation. Yet, optimal clinical management remains a mainstay of VA ECMO support. Therefore, it should be realized that LV overload during VA ECMO has an estimated incidence of up to 70% of cases and may significantly impact on survival.^6,35,71^ Clinically, LV overload may initially present as mildly elevated filling pressures, pulmonary edema and increased left-sided cardiac dimensions. Here, the initiation of an adjunct IABP can be considered, based on a large body of practice experience, when aiming to reduce a PCWP of 20 mmHg or less by maximally 5 mmHg^7,24^ (Figure 1b). When more potent unloading is required, a percutaneous Impella^®^ should be added to the VA ECMO as increasingly reported recently.^35,72^ The unloading effect of the Impella^®^ is comparable to more technically demanding percutaneous procedures, i.e. atrial septostomy and trans-septal left atrial venting and surgical unloading interventions^^7^^ (Figure 1b)(supplemental Table 1 and supplemental Figure 1). In this context, surgical adjunct interventions are especially opportune when patients undergo surgical intervention, i.e. thoracotomy or sternotomy, while the role of percutaneous pulmonary artery or trans-aortic LV venting is unclear, as the reported experience is very limited so far (supplemental Table 1 and supplemental Figure 1).

This LV overload situation may also deteriorate into a life-threatening accumulation of interrelated cardiopulmonary complications, cumulating in pulmonary hemorrhage,^65^ increasing spontaneous echo contrast “smoke” and LV thrombosis.^5,6,26–28,73^ An over-distended LV exposed to high myocardial stress, strain, work and oxygen consumption, as well as reduced coronary blood flow, will likely be unable to recover. Pulmonary edema may primarily occur as a consequence of high LV filling pressures, but systemic inflammation mediated by shock and impending multi-organ failure, as well as blood contact to artificial extracorporeal surfaces, may contribute. In this context, acute lung injury has been shown to significantly impact on prognosis in patients receiving VA ECMO, even after successful bridge-to-bridge therapy.^6^

Detailed clinical assessment of LV overload may be cumbersome, since its biomechanical impact on the failing myocardium may not be well-represented by standard clinical diagnostics.^3,4,71,74,75^ In general, repeated echocardiograms, assessment of intra-cardiac filling pressures and monitoring of pulmonary edema are imperative on a daily basis. Yet, the exact indication, timing and LV unloading strategy, i.e. percutaneous or surgical, should further be clarified in clinical studies. In addition, it has recently been demonstrated that the acute hemodynamic effects of all interventions detailed in this review can be simulated in computer models^2,4,7,76^ (Figure 1ab, Supplemental animation material - animation of heart failure with VA ECMO and unloading interventions). So far, these models, i.e. Aplysia CardioVascular Lab, Harvi and Basel Heart Simulator are scarce and have mainly been used for illustrations and educational purposes when simulating cardiac mechanical support modalities.^2,4,7,31,76^ These models await further clinical validation and it remains to be determined whether clinical decision-making in individual patients on VA ECMO can be improved by patient-specific cardiovascular computer modeling incorporating VA ECMO and other cardiac mechanical support modalities in real time and at the bedside, which is technically already possible.^2,4,7,76^

## Conclusion

VA ECMO can provide rapid and adequate cardiovascular support in patients with severe cardiogenic shock. Yet, the occurrence of LV overload during VA ECMO support frequently threatens its clinical success and should, therefore, be treated promptly. Here, we review the whole spectrum of adjunct percutaneous and surgical interventions that have successfully been used in clinical practice to unload the LV. It remains a clinical challenge to foresee which patients will benefit from adjunct interventions as they carry inherent procedural risks and it can be extremely cumbersome to predict the expected degree of LV unloading when intervening in an individual patient.

## Supplemental Material

Supplemental_Material_794112 – Supplemental material for Left ventricular unloading during veno-arterial ECMO: a review of percutaneous and surgical unloading interventions Click here for additional data file. Supplemental material, Supplemental_Material_794112 for Left ventricular unloading during veno-arterial ECMO: a review of percutaneous and surgical unloading interventions by Dirk W. Donker, Daniel Brodie, José P.S. Henriques and Michael Broomé in Perfusion
