# Supplementary material for: Left ventricular unloading during veno-arterial ECMO: a review of percutaneous and surgical unloading interventions
Source: Perfusion. 2018 Aug 16;34(2):98–105. doi: 10.1177/0267659118794112 (PMC6378398; doi:10.1177/0267659118794112)
Supplement: Supplemental_Material_794112 – Supplemental material for Left ventricular unloading during veno-arterial ECMO: a review of percutaneous and surgical unloading interventions [file Supplemental_Material_794112.pdf]

Left Ventricular Unloading during Veno-Arterial ECMO
A Review of Percutaneous and Surgical Unloading Interventions

Dirk W. Donker [*], Daniel Brodie [†], José P.S. Henriques [‡], Michael Broomé [§, ¶, &]

[*] Department of Intensive Care Medicine, University Medical Center Utrecht, Utrecht University, The Netherlands
[†] Division of Pulmonary, Allergy and Critical Care Medicine, Columbia University College of Physicians and Surgeons/New York-Presbyterian Hospital, New York, New York, USA
[‡] Department of Cardiology, Academic Medical Center, University of Amsterdam, Amsterdam, The Netherlands
[§] ECMO Department, Karolinska University Hospital, Stockholm, Sweden
[¶] Anaesthesiology and Intensive Care, Department of Physiology and Pharmacology, Karolinska Institute, Stockholm, Sweden
[&] School of Technology and Health, Royal Institute of Technology, Stockholm, Sweden

Corresponding author:

Michael Broomé MD PhD
ECMO Department
Karolinska University Hospital
SE-171 76 Stockholm
Sweden
michael.broome@ki.se

Supplemental material

Table 1. Summary of experience with percutaneous and surgical adjunct LV unloading interventions during VA ECMO as reported in the literature

Adjunct LV unloading intervention	Patient(s)		Setting	VA ECMO type	Weaning	Hospital mortality	Study type	Reference	Year	
IABP										
	1	adult	medical	peripheral	-	-	case report	[1]	2013	
	1	adult	post cardiotomy	central	-	-	case report	[2]	2013	
	2	adult	medical	peripheral	-	-	case series	[3]	2010	*
	2	adult	medical	peripheral	-	-	case series	[4]	2012	**
	3	adult	medical	peripheral	-	-	case series	[5]	2015	
	5	adult	medical	peripheral	-	-	case series	[6]	2012	**
	6	adult	medical	peripheral	50%	50%	case series	[7]	2005	*
	6	adult	post cardiotomy	peripheral	-	33%	case series	[8]	2009	
	6	adult	post cardiotomy/ post heart transplant/ medical	central/ peripheral	60%	55%	retrospective	[9]	2012	
	8	adult	post cardiotomy	central	-	-	case series	[10]	2011	
	11	adult	post cardiotomy/ medical	central/ peripheral	-	-	retrospective	[11]	2001	**
	11	adult	post cardiotomy/ medical	central/ peripheral	-	45%	retrospective	[12]	2011	*/ **
	12	adult	post cardiotomy/ post heart transplant/ medical	central/ peripheral	-	-	retrospective	[12]	2010	
	12	adult	medical	peripheral	25%	50%	prospective	[13]	2014	
	12	adult	post cardiotomy	peripheral	100%	33%	prospective	[14]	2014	
	13	adult	post cardiotomy/ medical	central/ peripheral	64%	64%	retrospective	[15]	2013	*
	13	adult	post heart transplant	central/ peripheral	84%	47%	retrospective	[16]	2014	
	14	adult	medical	peripheral	79%	50%	retrospective	[17]	2011	*
	20	adult	post cardiotomy/ medical	central/ peripheral	-	-	retrospective	[18]	2001	**
	19	adult	post cardiotomy	peripheral	30%	-	retrospective	[19]	2009	

21	adult	medical	peripheral	-	-	retrospective	[20]	2012	**
21	adult	medical	peripheral	43%	24%	retrospective	[21]	2013	
22	adult	post cardiotomy	central/ peripheral	-	68%	retrospective	[22]	2010	*/ **
27	adult	medical	peripheral	-	63%	retrospective	[23]	2013	*
30	adult	post cardiotomy/ post heart transplant/ medical	central/ peripheral	-	-	retrospective	[24]	2008	**
31	adult	post cardiotomy/ medical	peripheral	74%	42%	retrospective	[25]	2009	*
31	adult	medical	peripheral	71%	77%	retrospective	[26]	2006	*
35	adult	medical	peripheral	-	63%	retrospective	[27]	2014	*
37	adult	medical	central/ peripheral	-	-	retrospective	[28]	2011	**
38	adult	post cardiotomy	central/ peripheral	-	71%	retrospective	[29]	2017	
39	adult	post cardiotomy	central/ peripheral	15%	69%	retrospective	[30]	2013	*/ **
41	adult	post cardiotomy	peripheral	68%	32%	retrospective	[31]	2013	*
41	adult	medical	peripheral	63%	51%	retrospective	[32]	2014	*
51	adult	post cardiotomy/ post heart transplant	peripheral	53%	67%	retrospective	[33]	2010	
52	adult	medical	peripheral	-	-	retrospective	[34]	2010	**
54	adult	post cardiotomy/ post heart transplant/ medical	central/ peripheral	63%	61%	retrospective	[35]	2014	
60	adult	post cardiotomy/ medical	central/ peripheral	62%	67%	retrospective	[36]	2014	
72	adult	post cardiotomy	peripheral	-	69%	retrospective	[37]	2013	**
73	adult	post cardiotomy/ post heart transplant/ medical	peripheral	60%	55%	retrospective	[38]	2012	**
94	adult	medical	peripheral	55%	66%	retrospective	[39]	2012	*
95	adult	post cardiotomy	central/ peripheral	47%	71%	retrospective	[40]	2003	
135	adult	post cardiotomy/ medical	peripheral	41%	42%	retrospective	[41]	2015	
144	adult	post cardiotomy	central/ peripheral	60%	76%	retrospective	[42]	2004	
302	adult	post cardiotomy/ medical	peripheral	40%	48%	retrospective	[43]	2016	
383	adult	post cardiotomy	central/ peripheral	-	74%	retrospective	[44]	2010	*/ **
604	adult	medical	peripheral	83%	56%	retrospective	[45]	2016	

	844	adult	post cardiotomy/ medical	central/ peripheral	-	-	meta-analysis	[46]	2014
	1517	adult	post cardiotomy/ medical	central/ peripheral	-	65%	meta-analysis	[47]	2015
**Impella®**									
**2.5**	1	pediatric	medical	peripheral	-	-	case report	[48]	2006
**2.5**	1	adult	medical	peripheral	-	-	case report	[49]	2010
**2.5**	1	adult	medical	peripheral	-	-	case report	[50]	2011
**2.5**	1	adult	medical	peripheral	-	-	case report	[51]	2012
**2.5**	1	adult	medical	peripheral	-	-	case report	[52]	2012
**5.0**	1	adult	post cardiotomy	central	-	-	case report	[2]	2013
**2.5**	4	adult	medical	peripheral	-	50%	case series	[6]	2012
**2.5**	5	adult	medical	peripheral	-	20%	case series	[53]	2013
**CP**	6	adult	medical	peripheral	33%	33%	retrospective	[54]	2017
**2.5/ 5.0**	10	adult	post cardiotomy/ post heart transplant/ medical	central/ peripheral	50%	50%	retrospective	[55]	2017
**2.5/ CP**	12	adult	post cardiotomy/ post heart transplant/ medical	central/ peripheral	-	-	retrospective	[56]	2017
**5.0**	15	adult	post cardiotomy/ medical	peripheral	67%	-	retrospective	[57]	2015
**2.5**	21	adult	medical	peripheral	43%	24%	retrospective	[21]	2013
**2.5/ CP/ 5.0**	23	adult	post cardiotomy/ post heart transplant/ medical	central/ peripheral	-	-	retrospective	[58]	2017
**2.5/ 5.0**	27	adult	medical	peripheral	-	-	retrospective	[59]	2017
**2.5/ CP**	34	adult	medical	peripheral	48%	47%	retrospective	[60]	2016
**Percutaneous trans-septal venting**									
	1	pediatric	medical	peripheral	-	-	case report	[61]	1995
	1	pediatric	medical	peripheral	-	-	case report	[62]	2003
	1	pediatric	medical	peripheral	-	-	case report	[63]	2005
	1	pediatric	medical	peripheral	-	-	case report	[64]	2012
	1	adult	medical	peripheral	-	-	case report	[65]	2011
	1	adult	-	-	-	-	case report	[66]	2011

	1	adult	medical	peripheral	-	-	case report	67	2015
	1	pediatric	medical	peripheral	-	-	case report	68	2016
	4	adult	medical	peripheral			case series	69	2016
	7	adult/ pediatric	medical/ post heart transplant	-	57%	57%	case series	70	2006
	25	pediatric	medical	peripheral	-	-	case report	71	2015
	37	adult/ pediatric	post cardiotomy/ medical	-	57%	24%	retrospective	72	2015
	39	pediatric	medical/ post heart transplant	central/ peripheral	-	-	retrospective	73	2015
**Atrial septostomy**									
	1	pediatric	medical	peripheral	-	-	case report	74	1999
	1	adult	-	-	-	-	case report	75	2009
	1	adult	medical	peripheral	-	-	case report	76	2013
	1	adult	medical	peripheral	-	-	case report	77	2016
	1	pediatric	medical	peripheral	-	-	case report	78	2015
	1	adult	-	-	-	-	retrospective	79	2014
	1	adult	post cardiotomy/ post heart transplant/ medical	central/ peripheral	-	-	retrospective	56	2017
	4	neonatal	medical	peripheral	-	-	case series	80	1993
	7	pediatric	post cardiotomy/ medical	central/ peripheral	-	-	retrospective	81	2013
	5	pediatric	medical/ post heart transplant	central/ peripheral	-	-	retrospective	73	2015
	7	adult	medical	peripheral	71%	29%	case series	82	2017
	10	adult/ pediatric	medical	peripheral	40%	30%	retrospective	83	1999
	15	adult	medical	peripheral	40%	47%	case series	84	2017
	17	pediatric	medical	peripheral	-	-	case report	71	2015
	64	adult/ pediatric	post cardiotomy/ post heart transplant/ medical	peripheral	-	34%	retrospective	85	2016
**Pulmonary artery venting**									
	1	pediatric	medical	peripheral	-	-	case report	86	2011

	1	adult	medical	peripheral	-	-	case report	[87]	2011
	1	adult	medical	central/ peripheral	-	-	case report	[77]	2016
**Direct surgical PA venting**									
	1	pediatric	medical	central	-	-	case report	[88]	2013
	3	adult	post cardiotomy/ post heart transplant/ medical	central/ peripheral	-	-	retrospective	[58]	2017
**Trans-aortic venting**									
	1	adult	-	-	-	-	case report	[89]	2004
	1	adult	medical	peripheral	-	-	case report	[90]	2013
	1	adult	medical	peripheral	-	-	case series	[91]	2015
	3	adult	medical	peripheral	-	-	case series	[92]	2011
	7	adult	medical	peripheral	-	-	case series	[93]	2016
**Direct surgical LA venting**									
	1	adult	medical	peripheral	-	-	case report	[94]	2017
	2	adult	medical	central	-	-	case series	[95]	2014
	8	pediatric	post cardiotomy/ medical	central	88%	-	case series	[96]	2014
	9	adult	post cardiotomy/ post heart transplant/ medical	central/ peripheral	-	-	retrospective	[58]	2017
	16	pediatric	post cardiotomy/ medical	central/ peripheral	-	-	retrospective	[81]	2013
**Direct surgical LV venting**									
	1	pediatric	medical	peripheral	-	-	case report	[97]	2010
	1	adult	medical	central/ peripheral	-	-	case report	[98]	2013
	1	adult	medical	peripheral	-	-	case report	[99]	2015
	3	adult	medical	peripheral	-	-	case series	[100]	2011
	3	adult	post cardiotomy/ post heart transplant/ medical	central/ peripheral	-	-	retrospective	[56]	2017
	9	pediatric	medical/ post heart transplant	central/	-	-	retrospective	[73]	2015

			peripheral					
10	adult	post cardiotomy/ post heart transplant/ medical	central/ peripheral	-	-	retrospective	[58]	2017
12	adult	medical	central	25%	42%	prospective	[101]	2014

This summary has been created by full-text check of all primary, secondary and tertiary references and the 'similar articles' functionality in PubMed on https://www.ncbi.nlm.nih.gov/pubmed/ as cited in current reviews on LV unloading during VA ECMO [102-106], a recent meta-analysis on the role of the IABP during VA ECMO [47] and a recent meta-analysis on complications of VA ECMO [46].
LV indicates left ventricular, VA ECMO veno-arterial extracorporeal membrane oxygenation, Patient(s) indicates patients supported with VA ECMO and the adjunct LV unloading intervention indicated, Setting indicates the indication for VA ECMO with medical including cardiogenic shock due to myocardial infarction, myocarditis, dilated cardiomyopathy, etc. including cardiac arrest, VA ECMO type indicates the cannulation mode with peripheral including femoral vein-subclavian artery and carotid artery/ jugular vein cannulation, Weaning indicates the weaning rate reported, '-' indicates either that data are not representative, cannot be derived from the reference or are not provided. *Indicates studies included in a meta-analysis on the role of the IABP during VA ECMO [47] and **indicates studies included in a meta-analysis on complications in VA ECMO [46].

Figure 1. Overview of experience with percutaneous and surgical adjunct LV unloading interventions during VA ECMO as reported in the literature

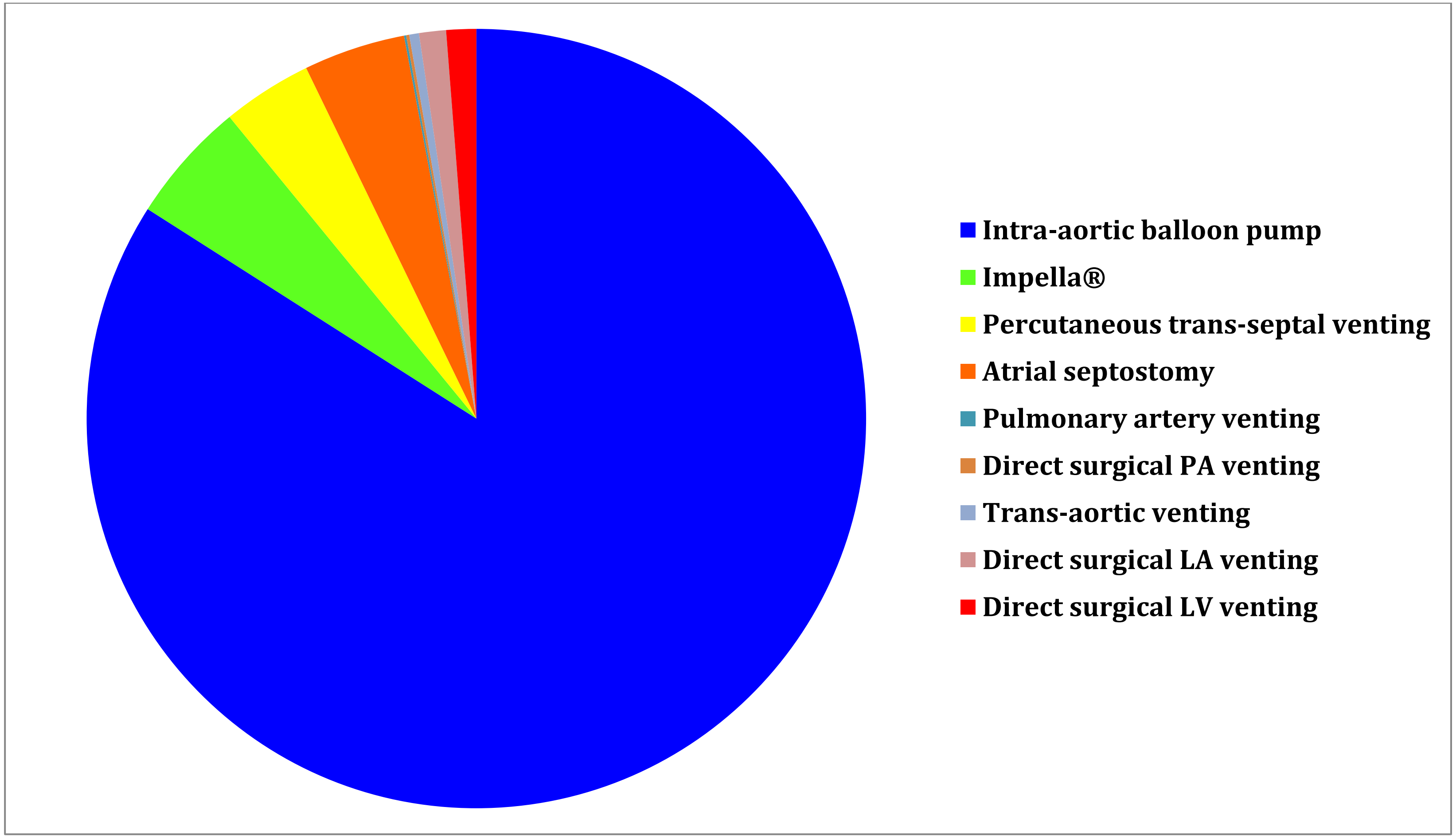

The intra-aortic balloon pump (IABP) is the most frequently reported LV unloading intervention during VA ECMO (n=2710 cases), with far less experience with Impella® (n=163 cases), percutaneous trans-septal venting (n=120 cases), atrial septostomy (n=136 cases), pulmonary artery (PA) venting (n=3 cases), direct surgical PA venting (n=4 cases), trans-aortic venting (n=13 cases), direct surgical venting of the left atrium (LA) (n= 36 cases) or left ventricle (LV) (n= 40 cases).

References

1. Delnoij TS, Wetzels AE, Weerwind PW, et al. Peripheral venoarterial extracorporeal life support despite impending left ventricular thrombosis: a bridge to resolution. *J Cardiothorac Vasc Anesth* 2013; 27: e48-49. DOI: 10.1053/j.jvca.2013.03.018.
2. Abu Saleh WK, Mason P, Jabbari OA, et al. Successful Use of Surgically Placed Impella 5.0 and Central Extracorporeal Membrane Oxygenation Circuit in a Patient with Postcardiotomy Shock. *Tex Heart Inst J* 2015; 42: 569-571. DOI: 10.14503/THIJ-14-4873.
3. Aziz TA, Singh G, Popjes E, et al. Initial experience with CentriMag extracorporal membrane oxygenation for support of critically ill patients with refractory cardiogenic shock. *J Heart Lung Transplant* 2010; 29: 66-71. DOI: 10.1016/j.healun.2009.08.025.
4. Kim H, Lim SH, Hong J, et al. Efficacy of veno-arterial extracorporeal membrane oxygenation in acute myocardial infarction with cardiogenic shock. *Resuscitation* 2012; 83: 971-975. DOI: 10.1016/j.resuscitation.2012.01.037.
5. Samadi B, Nguyen D, Rudham S, et al. Spinal Cord Infarct During Concomitant Circulatory Support With Intra-Aortic Balloon Pump and Veno-Arterial Extracorporeal Membrane Oxygenation. *Crit Care Med* 2016; 44: e101-105. DOI: 10.1097/CCM.0000000000001400.
6. Belle L, Mangin L, Bonnet H, et al. Emergency extracorporeal membrane oxygenation in a hospital without on-site cardiac surgical facilities. *EuroIntervention* 2012; 8: 375-382. DOI: 10.4244/EIJV8I3A57.
7. Asaumi Y, Yasuda S, Morii I, et al. Favourable clinical outcome in patients with cardiogenic shock due to fulminant myocarditis supported by percutaneous extracorporeal membrane oxygenation. *Eur Heart J* 2005; 26: 2185-2192. DOI: 10.1093/eurheartj/ehi411.
8. Madershahian N, Liakopoulos OJ, Wippermann J, et al. The impact of intraaortic balloon counterpulsation on bypass graft flow in patients with peripheral ECMO. *J Card Surg* 2009; 24: 265-268. DOI: 10.1111/j.1540-8191.2009.00807.x.
9. Loforte A, Murana G, Cefarelli M, et al. Role of Intra-Aortic Balloon Pump and Extracorporeal Membrane Oxygenation in Early Graft Failure After Cardiac Transplantation. *Artif Organs* 2016; 40: E136-145. DOI: 10.1111/aor.12793.
10. Madershahian N, Wippermann J, Liakopoulos O, et al. The acute effect of IABP-induced pulsatility on coronary vascular resistance and graft flow in critical ill patients during ECMO. *J Cardiovasc Surg (Torino)* 2011; 52: 411-418.
11. Smith C, Bellomo R, Raman JS, et al. An extracorporeal membrane oxygenation-based approach to cardiogenic shock in an older population. *Ann Thorac Surg* 2001; 71: 1421-1427.
12. Hei F, Lou S, Li J, et al. Five-year results of 121 consecutive patients treated with extracorporeal membrane oxygenation at Fu Wai Hospital. *Artif Organs* 2011; 35: 572-578. DOI: 10.1111/j.1525-1594.2010.01151.x.
13. Petroni T, Harrois A, Amour J, et al. Intra-aortic balloon pump effects on macrocirculation and microcirculation in cardiogenic shock patients supported by venoarterial extracorporeal membrane oxygenation*. *Crit Care Med* 2014; 42: 2075-2082. DOI: 10.1097/CCM.0000000000000410.

14. Yang F, Jia ZS, Xing JL, et al. Effects of intra-aortic balloon pump on cerebral blood flow during peripheral venoarterial extracorporeal membrane oxygenation support. *J Transl Med* 2014; 12: 106. DOI: 10.1186/1479-5876-12-106.
15. Mikus E, Tripodi A, Calvi S, et al. CentriMag venoarterial extracorporeal membrane oxygenation support as treatment for patients with refractory postcardiotomy cardiogenic shock. *ASAIO J* 2013; 59: 18-23. DOI: 10.1097/MAT.0b013e3182768b68.
16. Santise G, Panarello G, Ruperto C, et al. Extracorporeal membrane oxygenation for graft failure after heart transplantation: a multidisciplinary approach to maximize weaning rate. *Int J Artif Organs* 2014; 37: 706-714. DOI: 10.5301/ijao.5000353.
17. Chung ES, Lim C, Lee HY, et al. Results of Extracorporeal Membrane Oxygenation (ECMO) Support before Coronary Reperfusion in Cardiogenic Shock with Acute Myocardial Infarction. *Korean J Thorac Cardiovasc Surg* 2011; 44: 273-278. DOI: 10.5090/kjtcs.2011.44.4.273.
18. Pagani FD, Aaronson KD, Swaniker F, et al. The use of extracorporeal life support in adult patients with primary cardiac failure as a bridge to implantable left ventricular assist device. *Ann Thorac Surg* 2001; 71: S77-81; discussion S82-75.
19. Wang J, Han J, Jia Y, et al. Early and intermediate results of rescue extracorporeal membrane oxygenation in adult cardiogenic shock. *Ann Thorac Surg* 2009; 88: 1897-1903. DOI: 10.1016/j.athoracsur.2009.08.009.
20. Moraca RJ, Wanamaker KM, Bailey SH, et al. Salvage peripheral extracorporeal membrane oxygenation using Cobe Revolution((R)) centrifugal pump as a bridge to decision for acute refractory cardiogenic shock. *J Card Surg* 2012; 27: 521-527. DOI: 10.1111/j.1540-8191.2012.01467.x.
21. Tang GH, Malekan R, Kai M, et al. Peripheral venoarterial extracorporeal membrane oxygenation improves survival in myocardial infarction with cardiogenic shock. *J Thorac Cardiovasc Surg* 2013; 145: e32-33. DOI: 10.1016/j.jtcvs.2012.12.038.
22. Elsharkawy HA, Li L, Esa WA, et al. Outcome in patients who require venoarterial extracorporeal membrane oxygenation support after cardiac surgery. *J Cardiothorac Vasc Anesth* 2010; 24: 946-951. DOI: 10.1053/j.jvca.2010.03.020.
23. Beurtheret S, Mordant P, Paoletti X, et al. Emergency circulatory support in refractory cardiogenic shock patients in remote institutions: a pilot study (the cardiac-RESCUE program). *Eur Heart J* 2013; 34: 112-120. DOI: 10.1093/eurheartj/ehs081.
24. Bakhtiary F, Keller H, Dogan S, et al. Venoarterial extracorporeal membrane oxygenation for treatment of cardiogenic shock: clinical experiences in 45 adult patients. *J Thorac Cardiovasc Surg* 2008; 135: 382-388. DOI: 10.1016/j.jtcvs.2007.08.007.
25. Shinn SH, Lee YT, Sung K, et al. Efficacy of emergent percutaneous cardiopulmonary support in cardiac or respiratory failure: fight or flight? *Interact Cardiovasc Thorac Surg* 2009; 9: 269-273. DOI: 10.1510/icvts.2008.194860.
26. Chen JS, Ko WJ, Yu HY, et al. Analysis of the outcome for patients experiencing myocardial infarction and cardiopulmonary resuscitation refractory to conventional therapies necessitating extracorporeal life support rescue. *Crit Care Med* 2006; 34: 950-957. DOI: 10.1097/01.CCM.0000206103.35460.1F.
27. Aoyama N, Imai H, Kurosawa T, et al. Therapeutic strategy using extracorporeal life support, including appropriate indication, management, limitation and timing of switch to ventricular assist device in patients with acute myocardial infarction. *J Artif Organs* 2014; 17: 33-41. DOI: 10.1007/s10047-013-0735-z.

28. Bermudez CA, Rocha RV, Toyoda Y, et al. Extracorporeal membrane oxygenation for advanced refractory shock in acute and chronic cardiomyopathy. *Ann Thorac Surg* 2011; 92: 2125-2131. DOI: 10.1016/j.athoracsur.2011.07.029.
29. Biancari F, Dalen M, Perrotti A, et al. Venoarterial extracorporeal membrane oxygenation after coronary artery bypass grafting: Results of a multicenter study. *Int J Cardiol* 2017; 241: 109-114. DOI: 10.1016/j.ijcard.2017.03.120.
30. Unosawa S, Sezai A, Hata M, et al. Long-term outcomes of patients undergoing extracorporeal membrane oxygenation for refractory postcardiotomy cardiogenic shock. *Surg Today* 2013; 43: 264-270. DOI: 10.1007/s00595-012-0322-6.
31. Wang JG, Han J, Jia YX, et al. Outcome of veno-arterial extracorporeal membrane oxygenation for patients undergoing valvular surgery. *PLoS One* 2013; 8: e63924. DOI: 10.1371/journal.pone.0063924.
32. Park TK, Yang JH, Choi SH, et al. Clinical impact of intra-aortic balloon pump during extracorporeal life support in patients with acute myocardial infarction complicated by cardiogenic shock. *BMC Anesthesiol* 2014; 14: 27. DOI: 10.1186/1471-2253-14-27.
33. Hsu PS, Chen JL, Hong GJ, et al. Extracorporeal membrane oxygenation for refractory cardiogenic shock after cardiac surgery: predictors of early mortality and outcome from 51 adult patients. *Eur J Cardiothorac Surg* 2010; 37: 328-333. DOI: 10.1016/j.ejcts.2009.07.033.
34. Kagawa E, Inoue I, Kawagoe T, et al. Assessment of outcomes and differences between in- and out-of-hospital cardiac arrest patients treated with cardiopulmonary resuscitation using extracorporeal life support. *Resuscitation* 2010; 81: 968-973. DOI: 10.1016/j.resuscitation.2010.03.037.
35. Ma P, Zhang Z, Song T, et al. Combining ECMO with IABP for the treatment of critically Ill adult heart failure patients. *Heart Lung Circ* 2014; 23: 363-368. DOI: 10.1016/j.hlc.2013.10.081.
36. Ro SK, Kim JB, Jung SH, et al. Extracorporeal life support for cardiogenic shock: influence of concomitant intra-aortic balloon counterpulsation. *Eur J Cardiothorac Surg* 2014; 46: 186-192; discussion 192. DOI: 10.1093/ejcts/ezu005.
37. Slottosch I, Liakopoulos O, Kuhn E, et al. Outcomes after peripheral extracorporeal membrane oxygenation therapy for postcardiotomy cardiogenic shock: a single-center experience. *J Surg Res* 2013; 181: e47-55. DOI: 10.1016/j.jss.2012.07.030.
38. Loforte A, Montalto A, Ranocchi F, et al. Peripheral extracorporeal membrane oxygenation system as salvage treatment of patients with refractory cardiogenic shock: preliminary outcome evaluation. *Artif Organs* 2012; 36: E53-61. DOI: 10.1111/j.1525-1594.2011.01423.x.
39. Sakamoto S, Taniguchi N, Nakajima S, et al. Extracorporeal life support for cardiogenic shock or cardiac arrest due to acute coronary syndrome. *Ann Thorac Surg* 2012; 94: 1-7. DOI: 10.1016/j.athoracsur.2012.01.032.
40. Doll N, Fabricius A, Borger MA, et al. Temporary extracorporeal membrane oxygenation in patients with refractory postoperative cardiogenic shock--a single center experience. *J Card Surg* 2003; 18: 512-518.
41. Gass A, Palaniswamy C, Aronow WS, et al. Peripheral venoarterial extracorporeal membrane oxygenation in combination with intra-aortic balloon counterpulsation in patients with cardiovascular compromise. *Cardiology* 2014; 129: 137-143. DOI: 10.1159/000365138.

42. Doll N, Kiaii B, Borger M, et al. Five-year results of 219 consecutive patients treated with extracorporeal membrane oxygenation for refractory postoperative cardiogenic shock. *Ann Thorac Surg* 2004; 77: 151-157; discussion 157.
43. Lin LY, Liao CW, Wang CH, et al. Effects of Additional Intra-aortic Balloon Counter-Pulsation Therapy to Cardiogenic Shock Patients Supported by Extra-corporeal Membranous Oxygenation. *Sci Rep* 2016; 6: 23838. DOI: 10.1038/srep23838.
44. Rastan AJ, Dege A, Mohr M, et al. Early and late outcomes of 517 consecutive adult patients treated with extracorporeal membrane oxygenation for refractory postcardiotomy cardiogenic shock. *J Thorac Cardiovasc Surg* 2010; 139: 302-311, 311 e301. DOI: 10.1016/j.jtcvs.2009.10.043.
45. Aso S, Matsui H, Fushimi K, et al. The Effect of Intraaortic Balloon Pumping Under Venoarterial Extracorporeal Membrane Oxygenation on Mortality of Cardiogenic Patients: An Analysis Using a Nationwide Inpatient Database. *Crit Care Med* 2016; 44: 1974-1979. DOI: 10.1097/CCM.0000000000001828.
46. Cheng R, Hachamovitch R, Kittleson M, et al. Complications of extracorporeal membrane oxygenation for treatment of cardiogenic shock and cardiac arrest: a meta-analysis of 1,866 adult patients. *Ann Thorac Surg* 2014; 97: 610-616. DOI: 10.1016/j.athoracsur.2013.09.008.
47. Cheng R, Hachamovitch R, Makkar R, et al. Lack of Survival Benefit Found With Use of Intraaortic Balloon Pump in Extracorporeal Membrane Oxygenation: A Pooled Experience of 1517 Patients. *J Invasive Cardiol* 2015; 27: 453-458.
48. Vlasselaers D, Desmet M, Desmet L, et al. Ventricular unloading with a miniature axial flow pump in combination with extracorporeal membrane oxygenation. *Intensive Care Med* 2006; 32: 329-333. DOI: 10.1007/s00134-005-0016-2.
49. Jouan J, Grinda JM, Bricourt MO, et al. Successful left ventricular decompression following peripheral extracorporeal membrane oxygenation by percutaneous placement of a micro-axial flow pump. *J Heart Lung Transplant* 2010; 29: 135-136. DOI: 10.1016/j.healun.2009.06.007.
50. Koeckert MS, Jorde UP, Naka Y, et al. Impella LP 2.5 for left ventricular unloading during venoarterial extracorporeal membrane oxygenation support. *J Card Surg* 2011; 26: 666-668. DOI: 10.1111/j.1540-8191.2011.01338.x.
51. Chaparro SV, Badheka A, Marzouka GR, et al. Combined use of Impella left ventricular assist device and extracorporeal membrane oxygenation as a bridge to recovery in fulminant myocarditis. *ASAIO J* 2012; 58: 285-287. DOI: 10.1097/MAT.0b013e31824b1f70.
52. Narain S, Paparcuri G, Fuhrman TM, et al. Novel combination of impella and extra corporeal membrane oxygenation as a bridge to full recovery in fulminant myocarditis. *Case Rep Crit Care* 2012; 2012: 459296. DOI: 10.1155/2012/459296.
53. Cheng A, Swartz MF and Massey HT. Impella to unload the left ventricle during peripheral extracorporeal membrane oxygenation. *ASAIO J* 2013; 59: 533-536. DOI: 10.1097/MAT.0b013e31829f0e52.
54. Lim HS. The Effect of Impella CP on Cardiopulmonary Physiology During Venoarterial Extracorporeal Membrane Oxygenation Support. *Artif Organs* 2017. DOI: 10.1111/aor.12923.
55. Moazzami K, Dolmatova EV, Cocke TP, et al. Left Ventricular Mechanical Support with the Impella during Extracorporeal Membrane Oxygenation. *J Tehran Heart Cent* 2017; 12: 11-14.

56. Truby LK, Takeda K, Mauro C, et al. Incidence and Implications of Left Ventricular Distention During Venoarterial Extracorporeal Membrane Oxygenation Support. *ASAIO J* 2017; 63: 257-265. DOI: 10.1097/MAT.0000000000000553.
57. Gaudard P, Mourad M, Eliet J, et al. Management and outcome of patients supported with Impella 5.0 for refractory cardiogenic shock. *Crit Care* 2015; 19: 363. DOI: 10.1186/s13054-015-1073-8.
58. Tepper S, Masood MF, Baltazar Garcia M, et al. Left Ventricular Unloading by Impella Device Versus Surgical Vent During Extracorporeal Life Support. *Ann Thorac Surg* 2017; 104: 861-867. DOI: 10.1016/j.athoracsur.2016.12.049.
59. Eliet J, Gaudard P, Zeroual N, et al. Effect of Impella During Veno-Arterial Extracorporeal Membrane Oxygenation on Pulmonary Artery Flow as Assessed by End-Tidal Carbon Dioxide. *ASAIO J* 2017. DOI: 10.1097/MAT.0000000000000662.
60. Pappalardo F, Schulte C, Pieri M, et al. Concomitant implantation of Impella(R) on top of veno-arterial extracorporeal membrane oxygenation may improve survival of patients with cardiogenic shock. *Eur J Heart Fail* 2016. DOI: 10.1002/ejhf.668.
61. Ward KE, Tuggle DW, Gessouroun MR, et al. Transseptal decompression of the left heart during ECMO for severe myocarditis. *Ann Thorac Surg* 1995; 59: 749-751. DOI: 10.1016/0003-4975(94)00579-6.
62. Cheung MM, Goldman AP, Shekerdemian LS, et al. Percutaneous left ventricular "vent" insertion for left heart decompression during extracorporeal membrane oxygenation. *Pediatr Crit Care Med* 2003; 4: 447-449. DOI: 10.1097/01.PCC.0000075325.53339.CA.
63. Hlavacek AM, Atz AM, Bradley SM, et al. Left atrial decompression by percutaneous cannula placement while on extracorporeal membrane oxygenation. *J Thorac Cardiovasc Surg* 2005; 130: 595-596. DOI: 10.1016/j.jtcvs.2004.12.029.
64. Swartz MF, Smith F, Byrum CJ, et al. Transseptal catheter decompression of the left ventricle during extracorporeal membrane oxygenation. *Pediatr Cardiol* 2012; 33: 185-187. DOI: 10.1007/s00246-011-0113-7.
65. Kang MH, Hahn JY, Gwon HC, et al. Percutaneous transseptal left atrial drainage for decompression of the left heart in an adult patient during percutaneous cardiopulmonary support. *Korean Circ J* 2011; 41: 402-404. DOI: 10.4070/kcj.2011.41.7.402.
66. Madershahian N, Salehi-Gilani S, Naraghi H, et al. Biventricular decompression by trans-septal positioning of venous ECMO cannula through patent foramen ovale. *J Cardiovasc Surg (Torino)* 2011; 52: 900.
67. Jumean M, Pham DT and Kapur NK. Percutaneous bi-atrial extracorporeal membrane oxygenation for acute circulatory support in advanced heart failure. *Catheter Cardiovasc Interv* 2015; 85: 1097-1099. DOI: 10.1002/ccd.25791.
68. Kim HE, Jung JW, Shin YR, et al. Left Atrial Decompression by Percutaneous Left Atrial Venting Cannula Insertion during Venoarterial Extracorporeal Membrane Oxygenation Support. *Korean J Thorac Cardiovasc Surg* 2016; 49: 203-206. DOI: 10.5090/kjtcs.2016.49.3.203.
69. Alkhouli M, Narins CR, Lehoux J, et al. Percutaneous Decompression of the Left Ventricle in Cardiogenic Shock Patients on Venoarterial Extracorporeal Membrane Oxygenation. *J Card Surg* 2016; 31: 177-182. DOI: 10.1111/jocs.12696.

70. Aiyagari RM, Rocchini AP, Remenapp RT, et al. Decompression of the left atrium during extracorporeal membrane oxygenation using a transseptal cannula incorporated into the circuit. *Crit Care Med* 2006; 34: 2603-2606. DOI: 10.1097/01.CCM.0000239113.02836.F1.
71. Eastaugh LJ, Thiagarajan RR, Darst JR, et al. Percutaneous left atrial decompression in patients supported with extracorporeal membrane oxygenation for cardiac disease. *Pediatr Crit Care Med* 2015; 16: 59-65. DOI: 10.1097/PCC.0000000000000276.
72. O'Byrne ML, Glatz AC, Rossano JW, et al. Middle-term results of trans-catheter creation of atrial communication in patients receiving mechanical circulatory support. *Catheter Cardiovasc Interv* 2015; 85: 1189-1195. DOI: 10.1002/ccd.25824.
73. Hacking DF, Best D, d'Udekem Y, et al. Elective decompression of the left ventricle in pediatric patients may reduce the duration of venoarterial extracorporeal membrane oxygenation. *Artif Organs* 2015; 39: 319-326. DOI: 10.1111/aor.12390.
74. Johnston TA, Jaggers J, McGovern JJ, et al. Bedside transseptal balloon dilation atrial septostomy for decompression of the left heart during extracorporeal membrane oxygenation. *Catheter Cardiovasc Interv* 1999; 46: 197-199. DOI: 10.1002/(SICI)1522-726X(199902)46:2<197::AID-CCD17>3.0.CO;2-G.
75. Haynes S, Kerber RE, Johnson FL, et al. Left heart decompression by atrial stenting during extracorporeal membrane oxygenation. *Int J Artif Organs* 2009; 32: 240-242.
76. Peterss S, Pfeffer C, Reichelt A, et al. Extracorporeal life support and left ventricular unloading in a non-intubated patient as bridge to heart transplantation. *Int J Artif Organs* 2013; 36: 913-916. DOI: 10.5301/ijao.5000251.
77. Gultekin B, Ersoy O, Akkaya I, et al. Decompression of Left Ventricle During Venoarterial Extracorporeal Membrane Oxygenation Support as a Step to Transplant. *Exp Clin Transplant* 2016; 14: 42-44.
78. Veeram Reddy SR, Guleserian KJ and Nugent AW. Transcatheter removal of atrial septal stent placed to decompress left atrium with VA ECMO. *Catheter Cardiovasc Interv* 2015; 85: 1021-1025. DOI: 10.1002/ccd.25817.
79. Guenther S, Theiss HD, Fischer M, et al. Percutaneous extracorporeal life support for patients in therapy refractory cardiogenic shock: initial results of an interdisciplinary team. *Interact Cardiovasc Thorac Surg* 2014; 18: 283-291. DOI: 10.1093/icvts/ivt505.
80. Koenig PR, Ralston MA, Kimball TR, et al. Balloon atrial septostomy for left ventricular decompression in patients receiving extracorporeal membrane oxygenation for myocardial failure. *J Pediatr* 1993; 122: S95-99.
81. Kotani Y, Chetan D, Rodrigues W, et al. Left atrial decompression during venoarterial extracorporeal membrane oxygenation for left ventricular failure in children: current strategy and clinical outcomes. *Artif Organs* 2013; 37: 29-36. DOI: 10.1111/j.1525-1594.2012.01534.x.
82. Alhussein M, Osten M, Horlick E, et al. Percutaneous left atrial decompression in adults with refractory cardiogenic shock supported with veno-arterial extracorporeal membrane oxygenation. *J Card Surg* 2017; 32: 396-401. DOI: 10.1111/jocs.13146.
83. Seib PM, Faulkner SC, Erickson CC, et al. Blade and balloon atrial septostomy for left heart decompression in patients with severe ventricular dysfunction on extracorporeal membrane oxygenation. *Catheter Cardiovasc Interv* 1999; 46: 179-186. DOI: 10.1002/(SICI)1522-726X(199902)46:2<179::AID-CCD13>3.0.CO;2-W.

84. Lin YN, Chen YH, Wang HJ, et al. Atrial Septostomy for Left Atrial Decompression During Extracorporeal Membrane Oxygenation by Inoue Balloon Catheter. *Circ J* 2017; 81: 1419-1423. DOI: 10.1253/circj.CJ-16-1308.
85. Baruteau AE, Barnetche T, Morin L, et al. Percutaneous balloon atrial septostomy on top of venoarterial extracorporeal membrane oxygenation results in safe and effective left heart decompression. *Eur Heart J Acute Cardiovasc Care* 2016. DOI: 10.1177/2048872616675485.
86. Fouilloux V, Lebrun L, Mace L, et al. Extracorporeal membranous oxygenation and left atrial decompression: a fast and minimally invasive approach. *Ann Thorac Surg* 2011; 91: 1996-1997. DOI: 10.1016/j.athoracsur.2011.01.005.
87. Avalli L, Maggioni E, Sangalli F, et al. Percutaneous left-heart decompression during extracorporeal membrane oxygenation: an alternative to surgical and transeptal venting in adult patients. *ASAIO J* 2011; 57: 38-40. DOI: 10.1097/MAT.0b013e3181fe5d0b.
88. Kimura M, Kinoshita O, Fujimoto Y, et al. Central extracorporeal membrane oxygenation requiring pulmonary arterial venting after near-drowning. *Am J Emerg Med* 2014; 32: 197 e191-192. DOI: 10.1016/j.ajem.2013.09.031.
89. Fumagalli R, Bombino M, Borelli M, et al. Percutaneous bridge to heart transplantation by venoarterial ECMO and transaortic left ventricular venting. *Int J Artif Organs* 2004; 27: 410-413.
90. Chocron S, Perrotti A, Durst C, et al. Left ventricular venting through the right subclavian artery access during peripheral extracorporeal life support. *Interact Cardiovasc Thorac Surg* 2013; 17: 187-189. DOI: 10.1093/icvts/ivt119.
91. Hong TH, Byun JH, Yoo BH, et al. Successful Left-Heart Decompression during Extracorporeal Membrane Oxygenation in an Adult Patient by Percutaneous Transaortic Catheter Venting. *Korean J Thorac Cardiovasc Surg* 2015; 48: 210-213. DOI: 10.5090/kjtcs.2015.48.3.210.
92. Barbone A, Malvindi PG, Ferrara P, et al. Left ventricle unloading by percutaneous pigtail during extracorporeal membrane oxygenation. *Interact Cardiovasc Thorac Surg* 2011; 13: 293-295. DOI: 10.1510/icvts.2011.269795.
93. Hong TH, Byun JH, Lee HM, et al. Initial Experience of Transaortic Catheter Venting in Patients with Venoarterial Extracorporeal Membrane Oxygenation for Cardiogenic Shock. *ASAIO J* 2016; 62: 117-122. DOI: 10.1097/MAT.0000000000000327.
94. den Uil CA, Dos Reis Miranda D, Van Mieghem NM, et al. A Niche Indication for Intra-Aortic Balloon Pump Counterpulsation: Aortic Valve Opening in a Surgically Vented Left Ventricle on Venoarterial ECMO. *JACC Cardiovasc Interv* 2017; 10: e133-e134. DOI: 10.1016/j.jcin.2017.04.011.
95. Mohamedali B, Tatooles A and Bhat G. Use of a single circuit to provide temporary mechanical respiratory and circulatory support in patients with LV apical thrombus and cardiogenic shock. *Perfusion* 2014; 29: 483-487. DOI: 10.1177/0267659114538482.
96. Sandrio S, Springer W, Karck M, et al. Extracorporeal life support with an integrated left ventricular vent in children with a low cardiac output. *Cardiol Young* 2014; 24: 654-660. DOI: 10.1017/S1047951113001017.
97. Guirgis M, Kumar K, Menkis AH, et al. Minimally invasive left-heart decompression during venoarterial extracorporeal membrane oxygenation: an alternative to a percutaneous approach. *Interact Cardiovasc Thorac Surg* 2010; 10: 672-674. DOI: 10.1510/icvts.2009.228346.

98. Aggarwal A, Modi S, Kumar S, et al. Use of a single-circuit CentriMag(R) for biventricular support in postpartum cardiomyopathy. *Perfusion* 2013; 28: 156-159. DOI: 10.1177/0267659112464713.
99. Eudailey KW, Yi SY, Mongero LB, et al. Trans-diaphragmatic left ventricular venting during peripheral venous-arterial extracorporeal membrane oxygenation. *Perfusion* 2015; 30: 701-703. DOI: 10.1177/0267659115592468.
100. Rescigno GA, C.; Matteucci, M.L.; Massi, F.; Capestro, F.; D'Alfonso, A.; Torracca, L. Management of transapical left venting during adult peripheral extracorporeal membrane oxygenation. *Mechanical Circulatory Support* 2011; 2: 5981.
101. Weymann A, Schmack B, Sabashnikov A, et al. Central extracorporeal life support with left ventricular decompression for the treatment of refractory cardiogenic shock and lung failure. *J Cardiothorac Surg* 2014; 9: 60. DOI: 10.1186/1749-8090-9-60.
102. Rupprecht L, Florchinger B, Schopka S, et al. Cardiac decompression on extracorporeal life support: a review and discussion of the literature. *ASAIO J* 2013; 59: 547-553. DOI: 10.1097/MAT.0b013e3182a4b2f6.
103. Soleimani B and Pae WE. Management of left ventricular distension during peripheral extracorporeal membrane oxygenation for cardiogenic shock. *Perfusion* 2012; 27: 326-331. DOI: 10.1177/0267659112443722.
104. Strunina SO, P. Left ventricle unloading during veno-arterial extracorporeal membrane oxygenation. *Curr Res Cardio* 2016; 3: 5-8.
105. Greco GC, B.; Avalli, L. Left Ventricular Rest and Unloading During VA ECMO. *ECMO-Extracorporeal Life Support in Adults* 2014: 193-206. DOI: DOI 10.1007/978-88-470-5427-1_17.
106. Meani P, Gelsomino S, Natour E, et al. Modalities and Effects of Left Ventricle Unloading on Extracorporeal Life support: a Review of the Current Literature. *Eur J Heart Fail* 2017; 19 Suppl 2: 84-91. DOI: 10.1002/ejhf.850.
